# CTAB-Modulated Electroplating of Copper Micropillar Arrays for Non-Enzymatic Glucose Sensing with Improved Sensitivity

**DOI:** 10.3390/s24051603

**Published:** 2024-02-29

**Authors:** Wenhao Yao, Hu He, Fuliang Wang

**Affiliations:** State Key Laboratory of Precision Manufacturing for Extreme Service Performance, College of Mechanical and Electrical Engineering, Central South University, Changsha 410083, China; 213701008@csu.edu.cn

**Keywords:** electrochemical detection, copper deposition, cetyltrimethylammonium bromide

## Abstract

Micropillar array electrodes represent a promising avenue for enhancing detection sensitivity and response current. However, existing methods for depositing electrode materials on micropillar arrays often result in uneven distribution, with the thin sidewall layer being less conductive and prone to corrosion. In addressing this issue, this study introduces electroplating to enhance the copper layer on the sidewall of micropillar array electrodes. These electrodes, fabricated through standard microelectronics processes and electroplating, are proposed for non-enzymatic glucose detection, with the copper layer deposited via electroplating significantly enhancing sensitivity. Initially, the impact of cetyltrimethylammonium bromide (CTAB) concentration as an inhibitor on the surface morphology and sensitivity of the plated layer was investigated. It was discovered that CTAB could decrease surface roughness, hinder the development of large and coarse grains, generate small particles, and boost sensitivity. Compared to the uncoated electrode and plating without CTAB, sensitivity was elevated by a factor of 1.66 and 1.62, respectively. Subsequently, the alterations in plating morphology and detection performance within a range of 0.3 ASD to 3 ASD were examined. Sensitivity demonstrated a tendency to increase initially and then decrease. The electrode plated at 0.75 ASD achieved a maximum sensitivity of 3314 μA·mM^−1^·cm^−2^ and a detection limit of 15.9 μM. Furthermore, a potential mechanism explaining the impact of different morphology on detection performance due to CTAB and current density was discussed. It was believed that the presented effective strategy to enhance the sensitivity of micropillar array electrodes for glucose detection would promote the related biomedical detection applications.

## 1. Introduction

With over 500 million individuals worldwide affected by diabetes, the demand for accurate, sensitive, convenient, and cost-effective glucose detection techniques is paramount [1]. Among the various available methods, electrochemical sensors have gained significant attention due to their inherent advantages of speed, simplicity, selectivity, and affordability, making them promising alternatives to conventional approaches [2]. As the primary direction in this field, non-enzymatic electrochemical sensors have further addressed the limitations of enzyme-based sensors, such as high cost, complex fabrication procedures, and limited shelf life [2,3]. Recent research has focused on utilizing copper-based materials in non-enzymatic electrochemical sensors due to their natural abundance, low cost, non-toxicity, and high catalytic activity [4]. The efficacy of copper-based materials in enhancing glucose detection has been substantiated by various studies. Yogesh et al. [5] fabricated a non-enzymatic glucose sensor by copper nanowire–carbon nanotube bilayer (Cu-NW-CNT-BL) on a glassy carbon (GC) electrode, the sensitivity of which amounted to 1907 μA mM^−1^ cm^−2^. Wu et al. [6] developed a copper oxide–carboxylated graphene nanocrystal-modified electrode by utilizing electrodeposition and electrochemical oxidization. The electrode exhibited great detection ability with a sensitivity of 1295 μA mM^−1^ cm^−2^ and linear range from 0.1 μM to 3.17 mM. Chahira et al. [7] developed an effective electrochemical glucose sensor by electrodepositing copper dendrites hydroxide on to a pencil graphite electrode (Cu(OH)_2_/PGE). The sensitivity and the detection limit of the electrode was found to be 1064.7 μA mM^−1^ cm^−2^ and 0.2 μM, when it was evaluated by amperometry. The modification of conventional planar electrodes with these materials has yielded sensors characterized by low detection limits, wide linear ranges, and high sensitivity.

While previous studies predominantly employed conventional planar electrodes, novel techniques such as microelectrode arrays and micropillar array electrodes have emerged to further enhance detection performance. Within a specific projection area, micropillar array electrodes offer a significantly larger surface area compared to planar electrodes due to their three-dimensional structure [8,9]. This expanded surface area allows micropillar array electrodes to interact with a larger volume of analytes, leading to enhanced current responses and sensitivities [10,11]. Applications of micropillar array electrodes span various fields, showcasing their performance advantages over planar electrodes. Chang et al. successfully fabricated flexible micropillar electrodes composed of a polymer and carbon nanotube composite, enabling the detection of dopamine via differential pulse voltammetry [12]. Movilli et al. designed gold-coated silicon micropillar-structured electrodes for DNA detection, demonstrating a substantial enhancement in electrode area and current response by modifying the pillar array pitch [13]. Dervisevic et al. developed a wearable sensor for sweat glucose detection [14]. The chitosan–Au nanoparticle (Ch-AuNP) and glucose oxidase (GOx) modified sensor has a linear range of 50 μM to 1.4 mM and a sensitivity of 4.7 ± 0.8 μA mM^−1^. Chen et al. fabricated micropillar array electrodes modified with different materials to detect hydrogen peroxide and sarcosine [15]. The micropillar array electrodes demonstrated a 1.5 times larger sensitivity compared with the planar electrode when hydrogen peroxide was detected. Furthermore, they developed a microfluidic device integrated with polymethyl methacrylate (PMMA)-based micropillar array electrodes to detect multiple biomarkers [10]. The GOx-modified electrode exhibits a linear response to glucose from 0.1 mM to 12 mM, and the limit of detection (LOD) is 58.5 μM.

This work aims to develop a high-sensitivity non-enzymatic sensor by employing copper and micropillar array electrodes. Universal membrane deposition techniques are employed for electrode material manufacturing on micropillar arrays. However, challenges arise from the uneven distribution of the copper layer deposited on micropillar array electrodes through deposition techniques, particularly with a thinner layer on the sidewalls. This thin layer results in poor electrical conductivity and increased susceptibility to corrosion. To address this issue, electrochemical deposition is introduced after the magnetron sputtering process to enhance the copper layer. Achieving uniformly distributed and morphologically controllable layers often necessitates the inclusion of additives in plating solutions. CTAB emerges as a crucial additive for Cu plating, demonstrating its efficacy in attaining uniform and controllable deposition across various plating solutions and substrates [16,17,18]. Hence, CTAB is employed to control the plated layer morphology and enhance deposition uniformity. The study evaluates the morphology and sensitivities of the plated layers under different additive concentrations and current densities, proposing a potential mechanism to explain the impact of varying morphologies on detection sensitivities due to CTAB concentrations and current densities.

## 2. Materials and Methods

### 2.1. Materials and Chemicals

The silicon wafer was n-type (100), resistivity < 0.01 Ω·cm, 550 μm thick, purchased from Henan Micro-Nano Semiconductor Company. AZ4620 photoresist, developer, and magnetron targets were also purchased from the company. Sodium chloride (99.7%), sodium hydroxide (95%), glucose (99.7%), concentrated sulfuric acid (98%), and CTAB (99%) were purchased from Sinopharm. Copper sulfate pentahydrate (99.7%), sucrose (99.9%), ascorbic acid (AA, >99.0%), and uric acid (UA, 99%) were purchased from Macklin. Fructose (99%) provided by Aladdin. Deionized water was self-made in the lab.

### 2.2. Fabrication Process and Electrode Structure of Silicon Pillar Array Electrodes

**Figure 1:**
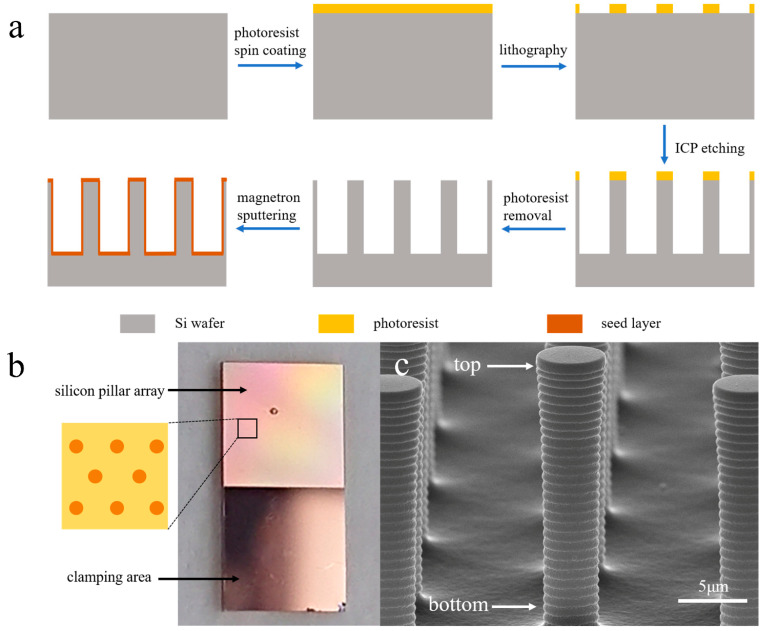
Schematic of silicon pillar array fabrication process (**a**), structural representation of the electrode (**b**), SEM image of the silicon pillar array (**c**).

Figure 1a illustrates the schematic of the fabrication process for the silicon pillar array electrodes. Initially, silicon pillar arrays with a length of 20 μm were created from the wafer using photolithography and inductively coupled plasma (ICP) etching. Following the removal of the photoresist, the wafer underwent a thorough cleaning process. Subsequently, a seed layer was deposited on the wafer using magnetron sputtering, comprising a 1 μm Cu layer over a 30 nm Ti layer. Prior to wafer dicing, an additional layer of photoresist was applied to safeguard the pillar arrays. The wafer was then cut into 5 mm × 10 mm chips, serving as electrodes. The testing area, measuring 5 mm × 5 mm, featured a silicon pillar array, while the opposite side was designated for clamping purposes. The pillars within the array were arranged in a hexagonal close-packing configuration, with a center-to-center distance of 15 μm. In accordance with prior research [8,9], the dense array arrangement and high aspect ratio may give rise to an array structure system characterized by overlapping diffusion layers.

A graphical representation of the electrode structure is depicted in Figure 1b, and the SEM image in Figure 1c provides further insight into the silicon pillar array, where the top diameter of the pillar measures 4.70 μm, and the bottom diameter is 3.49 μm.

### 2.3. Experimental Setup for Electroplating Process of Silicon Pillar Array Electrodes

**Figure 2:**
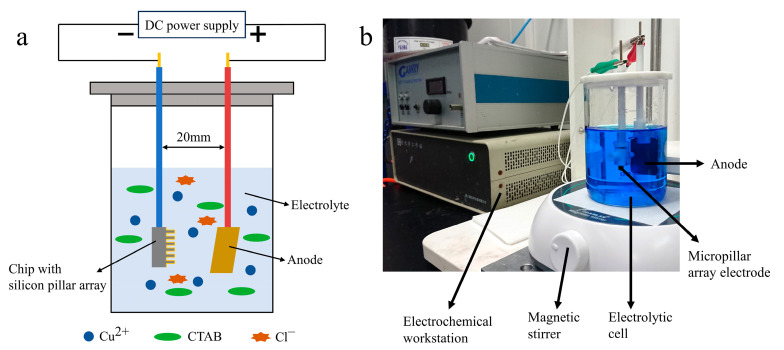
Schematic of experimental setup of electroplating (**a**), configuration of the electroplating setup (**b**).

Figure 2 illustrates the schematic of the experimental setup. During the plating process, the chip served as the cathode and was connected to the electrochemical workstation (CHI 660E, Chen Hua Instrument, Shanghai, China). The anode, a copper plate measuring 30 mm × 25 mm × 1 mm, was positioned at a distance of 20 mm from the cathode. Plating was carried out in a 250 mL electrolytic cell with a magnetic stirrer rotating at 350 rpm to ensure uniform solution mixing. 

As a widely used inhibitor, CTAB can effectively suppress stray and unevenly distributed crystals on the plated layer. To investigate the impact of CTAB concentration on the morphology and detection performance of the plated layers, various concentrations of CTAB (0, 0.01, 0.1, and 1 g/L) were explored. The electrolyte composition, in addition to CTAB, comprised 250 g of CuSO_4_·5H_2_O, 0.1 g of NaCl, and 32 mL of H_2_SO_4_ (98%) per liter. As a control, bare electrodes were also included in the study. The experiments were conducted at a fixed current density of 0.3 A dm^−2^ (ASD) for a duration of 1 h.

Furthermore, the influence of current density on the morphology and detection performance of the plated layers was explored. To regulate the deposition mass and prevent overfilling of the pillar arrays, a fixed electric charge of 4.5 C was maintained. This was achieved by varying the plating times to 100, 40, 20, and 10 min, corresponding to current densities of 0.3, 0.75, 1.5, and 3 ASD, respectively.

### 2.4. Electrode Surface Morphology Characterization and Glucose Detection Experiments

The morphology of the electrodes was elucidated using scanning electron microscopy (SEM, Tescan, VEGA3, Brno, Czech Republic). To enable a comprehensive view of the sample’s morphology, both the top and bottom were simultaneously observed by tilting them at an angle of 15°.

Glucose detection experiments were conducted in a 10 mL electrolytic cell. The back of the silicon chips was covered with PVC tape to eliminate interferences. During the chronoamperometry (CA), glucose was added into a 0.1 M NaOH solution every 50 s, and the solution was continuously stirred. Details of the tests including concentrations, volumes, and frequencies are shown in Table 1. Cyclic voltammetry (CV) tests were carried out in both 0.1 M NaOH solution and NaOH solution with 5 mM glucose solution. The scanning rates used were 40, 60, 80, 100, 120, 140, and 160 mV/s.

## 3. Result and Discussion

### 3.1. Influence of CTAB Concentration on Electroplating Morphology

**Figure 3:**
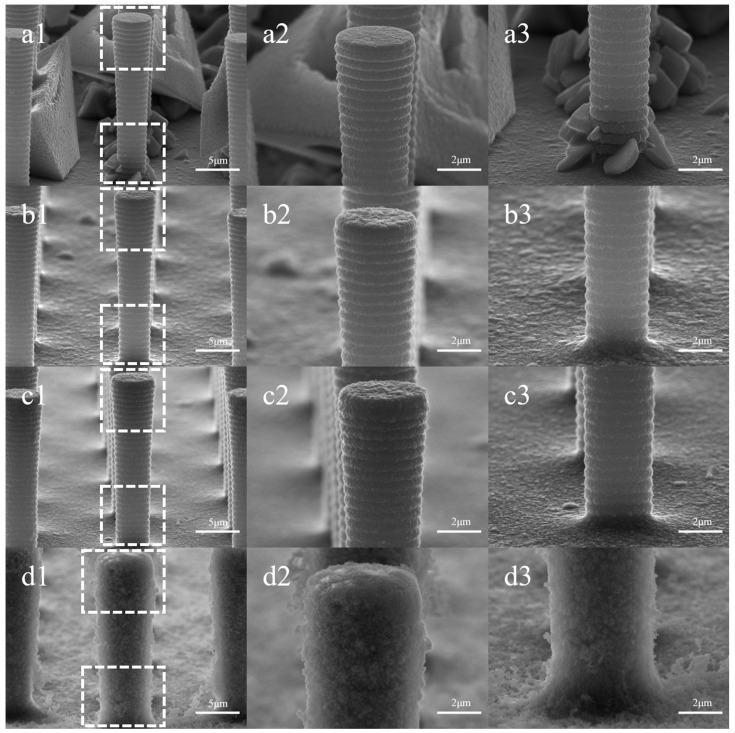
Surface morphology with varied CTAB concentrations: (**a1**–**a3**) for 0 g/L, (**b1**–**b3**) for 0.01 g/L, (**c1**–**c3**) for 0.1 g/L, (**d1**–**d3**) for 1 g/L. Columns 2 and 3 represent localized magnifications of the top and bottom of the silicon pillar in column 1, respectively.

Figure 3 shows the morphological changes in the electrodes plated with varying concentrations of CTAB. In Figure 3(a1–a3), Cu deposition exhibits needle-like, tetrahedral, or polyhedral crystal structures, primarily growing at the base of the pillars, forming clusters or larger tetrahedrons partially encasing the pillars. In contrast, the surface texture of the top, side wall, and bottom of the Si pillars closely resembles that of the bare electrode, maintaining a flat appearance. Figure 3(b1–b3) demonstrates that the presence of CTAB suppresses the copper deposition at the bottom of the pillars. The addition of 0.01 g/L of CTAB changes the deposition pattern of copper from crystal formations to plating with protrusions. Plated structures begin to appear at the top of the silicon pillars while leaving the diameter nearly unchanged. In Figure 3(c1–c3), the suppression of protrusions at the bottom results in a smoother plating, with the top pillar diameter measuring 5.24 μm and the bottom diameter at 3.97 μm. With a further increase in CTAB concentration, a velvety texture appears on the surface of the pillars. The sidewalls are entirely covered, and the original texture becomes indiscernible. At this stage, the top diameter reaches 7.04 μm, and the bottom diameter is 5.95 μm.

The influence of CTAB on copper deposition morphology can be attributed to its adsorption behavior and interactions with copper ions. By adsorbing to the surface of the silicon pillars, CTAB reduces the local electric field difference, effectively suppressing the growth of large, protruding crystals and promoting a more uniform plating layer. The wide spacing of 10.3 μm between the silicon pillars and their aspect ratio of less than 1:2 facilitate the diffusion of CTAB molecules to the bottom of the pillars, ensuring effective inhibition of bulk crystal growth across the entire electrode surface.

At lower CTAB concentrations, the minimal number of adsorbed CTAB molecules on the pillar array surface results in a weak inhibitory effect. Consequently, the plating layer tends to level out, as observed in Figure 3(c1–c3). As the CTAB concentration increases, more molecules are adsorbed, leading to a stronger inhibitory effect and the formation of a smoother layer without significant protrusions.

**Figure 4:**
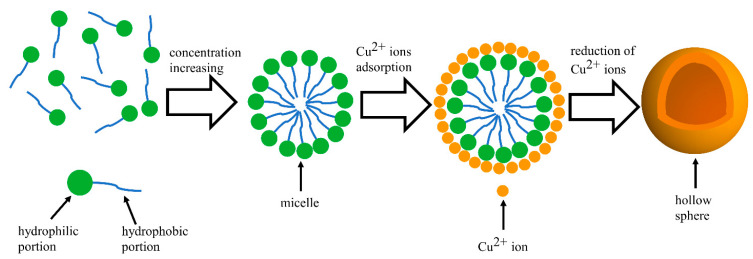
Schematic representation of the formation process of micelles and hollow spherical particles.

However, at high CTAB concentrations, the strong affinity of the hydrophilic portion of CTAB molecules induces aggregation, forming micellar clusters. This aggregation is characterized by the critical micelle concentration, approximately 0.33 g/L for CTAB [19,20,21]. The Br^−^ of CTAB molecules can capture Cu^2+^ ions through electrostatic interaction, which results in an enrichment of Cu^2+^ at the interface between the CTAB micelle and the aqueous solution [22]. The Cu^2+^ ions were then reduced, possibly leading to the formation of hollow particles that replicate the shape of the micelles. A schematic of the process is shown in Figure 4. The accumulation of these particles on the electrode surface gives rise to the velvety texture and expanded thickness seen in Figure 3(d1–d3).

### 3.2. The Impact of CTAB on the Performance of Copper-Plated Electrodes for Glucose Sensing

**Figure 5:**
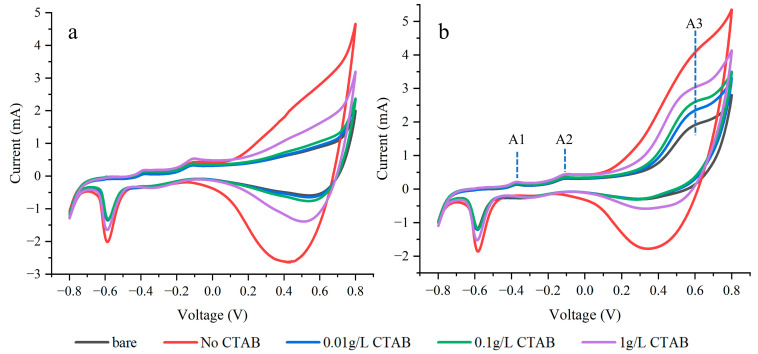
CV curves in 0.1 M NaOH (**a**) and NaOH solution with 5 mM glucose (**b**) at 100 mV/s.

Figure 5 presents the CV test curves for electrodes plated with varying concentrations of CTAB. A prominent enhancement in the response of all electrodes is observed at +0.6 V in the presence of glucose. During the forward scan, three oxidation peaks labeled A1, A2, and A3 sequentially emerge. The possible reaction process and mechanisms of these three oxidation peaks have been reported [23,24,25]. At the A1 peak, Cu(0) undergoes oxidation, forming Cu(I) and leading to the creation of Cu_2_O. Subsequently, Cu(I) is further oxidized to Cu(II), resulting in the formation of either CuO at the A2 peak. At the potential of +0.6 V, Cu(II) is oxidized to Cu(III), forming CuO(OH). The redox reaction between Cu(III) and glucose, involving electron transfer, amplifies the current response, leading to the appearance of the A3 peak, which is used for glucose detection.

The reactions occurring at each oxidation peak are as follows:

A1:

$$2Cu+2{OH}^{-}\to {Cu}_{2}O+H_{2}O+2e^{-}$$

A2:

$${Cu}_{2}O+2{OH}^{-}\to 2CuO+H_{2}O+2e^{-}$$

A3:

$$CuO+{OH}^{-}\to CuO(OH)+e^{-}$$

$$CuO\left(OH\right)+Glucose\to CuO+Gluconolactone$$

Gluconolactone→Gluconic Acid

**Figure 6:**
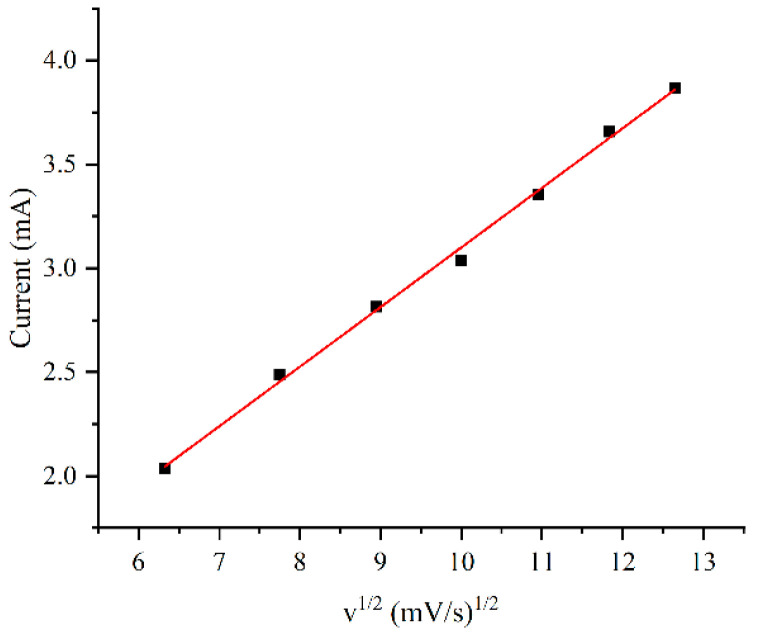
Oxidation peak current in 5 mM glucose solution as a function of scan rate for the electrode plated with 1 g/L CTAB.

The influence of scan rate on the peak current was also investigated, as shown in Figure 6. When the structure was plated using a solution containing 1 g/L of CTAB, the oxidation peak current exhibited a linear response to the square root of the scan rate. The relationship indicates that the oxidation process at the electrode is a typical diffusion-controlled process.

**Figure 7:**
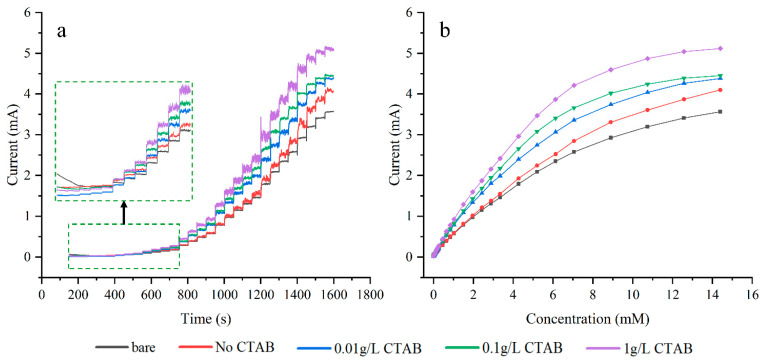
CA plots of electrodes with varied plating solution concentrations (**a**), response curves at different glucose concentrations (**b**).

Figure 7a illustrates the amperometric response of different electrodes at +0.60 V with successive additions of glucose. It can be observed that the current response rises as the concentration of CTAB increases. The electrode plated with 1 g/L CTAB exhibits the largest current response, reaching the maximum current density of 20.48 mA/cm^2^. In Figure 7b, when the concentration of glucose reaches 10 mM or more, all the response curves turn downward. The currents gradually reach saturation values at high concentrations, suggesting that the active sites of Cu are saturated at these glucose levels. 

**Figure 8:**
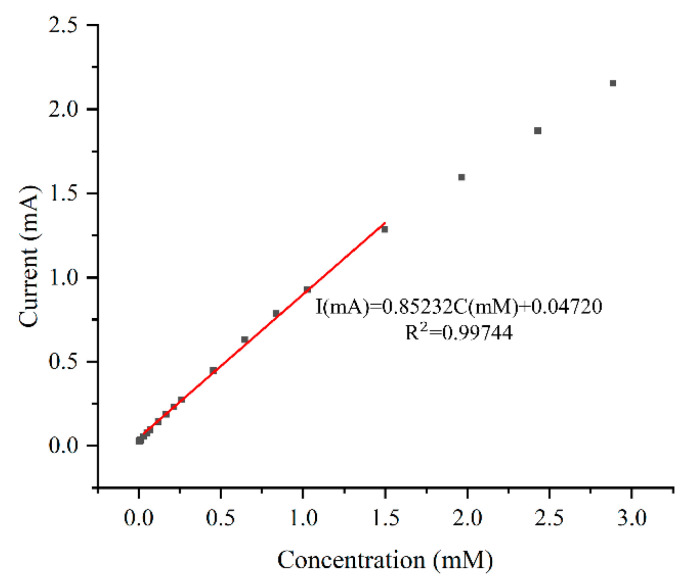
Linear fitting for sample plated with 1 g/L CTAB solution.

The data range of the regression equations for all electrodes is from 0.03 mM to 1.5 mM. The sensitivities of all electrodes follow a similar trend as the response curves in Figure 7. The bare electrode has the lowest sensitivity of 2059 μA mM^−1^ cm^−2^. Plating without CTAB slightly increases sensitivity by 2%. As the concentration of CTAB increases, so does the sensitivity, with the electrode plated with 1 g/L CTAB showing the maximum sensitivity of 3409 μA mM^−1^ cm^−2^. This is 1.66 and 1.62 times that of the bare electrode and the plating without CTAB case, respectively. The calibration curve for the electrode plated with 1 g/L CTAB is depicted in Figure 8.

In the absence of CTAB, Cu is deposited as large crystals accumulating at the bottom of the array. Compared to the bare electrode, the added macroscale crystal structures increase the surface area of the electrode and exhibit good electrical properties. As a result, the electrode shows the maximum CV currents in Figure 5a,b and contributes to a higher blank current in Figure 7a.

The addition of CTAB alters the surface and structures of the copper plating. At a concentration of 1 g/L, the particles and protrusions on the plated surface reach the nanometer scale. The electrochemical and catalytic properties become more active as the scale of the surface structure shrinks to nanostructures. Additionally, the diameter at the top of the silicon pillar increased from 4.89 μm at 0.01 g/L up to 7.04 μm at 1 g/L. The presence of micelles not only refines the grains further, but also augments the diameter of pillars and surface area of the array. Consequently, in Figure 7a, when an equivalent amount of glucose is added, electrodes treated with higher CTAB concentrations exhibit a greater increment in current response and sensitivity. It is important to note that the observed performance enhancement, when compared to lower CTAB concentrations, may be influenced by both factors.

### 3.3. Impact of Current Density on the Morphology of Electroplated Copper Layers

Previous studies have demonstrated that electrodes plated with a high concentration of CTAB exhibit superior sensitivity. In addition, the inhibitory effect of CTAB weakens significantly at higher potentials [26]. Consequently, the concentration of CTAB was consistently maintained at 1 g/L.

**Figure 9:**
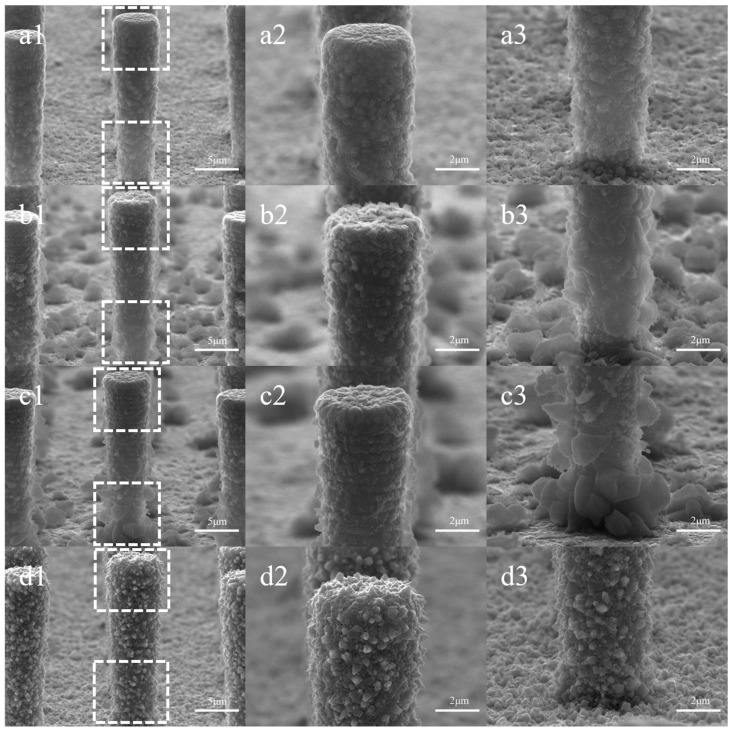
Surface morphology at different current densities: (**a1**–**a3**) for 0.3 ASD, (**b1**–**b3**) for 0.75 ASD, (**c1**–**c3**) for 1.5 ASD, (**d1**–**d3**) for 3 ASD. Columns 2 and 3 depict localized magnifications of the top and bottom of the silicon pillar in column 1, respectively.

Figure 9 illustrates the morphology of silicon pillars plated at different current densities and their local magnification. In Figure 9(a1–a3), the morphology of the plated layer aligns with the same current density in Figure 3(d1–d3), showing minimal impact from the increase in plating time. However, as the current density increases to 0.75 ASD and 1.5 ASD, noticeable large and coarse particles emerge at the bottom of the array and the sidewalls of the silicon pillars. On the sidewalls, the particles near the bottom of the pillars exhibit larger diameters, with some exceeding 2 μm. In the local magnification of the top of the silicon pillars, the particles form a circular texture along the surface. When the current density reaches 3 ASD, all surfaces of the arrays and silicon pillars become coated with coarse particles, mostly with heights less than 500 nm, and some adopting a sharp conical shape.

The morphological changes of the plating layer should be considered in conjunction with the inhibitory effect of CTAB and the influence of current density on deposition. Under low current density, the inhibitory effect of CTAB is pronounced. On the silicon pillar surface, the layer does not exhibit large crystalline particles, and the diameter of the protruding particles at the bottom of the array is small. At higher current density, the inhibitory effect of CTAB diminishes, and the impact of current density becomes more pronounced. The observed variations in the morphology of electroplated Cu layers align with the existing literature [27,28]. According to the principles of electrocrystallization, the rate of formation of new atomic nuclei on the electrode surface, ω, undergoes exponential growth with increasing cathodic polarization, η [29,30]:

$$\omega =B\;\exp(-\frac{K}{\eta^{2}})$$

where the constants B and K are specific for a given metal and temperature. Under unchanged conditions, an increase in current density or voltage results in a larger cathodic overpotential. This implies a significant enhancement in the nucleation rate on the electrode surface, leading to the formation of a smoother coating. At high current density (3 ASD), the appearance of sharp grains on the layer surface may be correlated with the distribution of the electric field and the surface tension of the solution [30]. When the deposit plane is oriented as normal to the electric field, its growth direction is symmetrical to the field direction. Additionally, the presence of CTAB reduces the surface tension of the electrolyte, potentially facilitating grain growth along the direction of the high electric field.

### 3.4. Influence of Current Density on the Performance of Copper-Plated Electrode Arrays for Glucose Sensing

**Figure 10:**
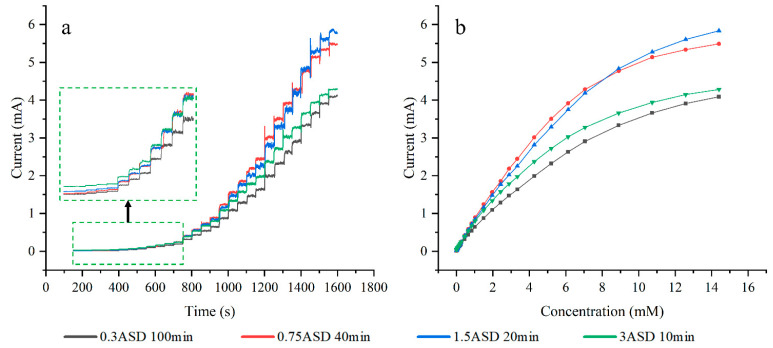
CA plots of electrodes plated at different current densities (**a**), response curves of each electrode at different glucose concentrations (**b**).

Figure 10a presents typical amperometric responses of glucose in 0.1 M NaOH at electrodes plated with different current densities. Among all samples, the electrode plated at 0.75 ASD exhibits the highest current response across most measured concentrations. This electrode attains a response current of 5.489 mA and a current density of 21.956 mA/cm^2^. The current responses of electrodes plated with either higher or lower current densities show significant decreases. In Figure 10b, the current response of the electrode plated at 1.5 ASD surpasses that of the 0.75 ASD-coated electrode when the glucose concentration exceeds 8.9 mM.

**Figure 11:**
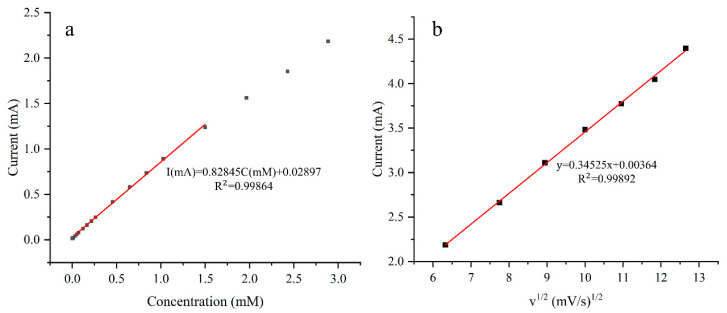
Linear fitting of a sample plated at 0.75 ASD (**a**), and oxidation peak current in 5 mM glucose solution as a function of scan rate (**b**).

The electrode plated at 0.75 ASD achieves an optimized sensitivity of 3314 μA mM^−1^ cm^−2^, which is 1.417 times greater than the minimum sensitivity. The detection limit of the electrode is 15.9 μM, and the calibration curve of the electrode is shown in Figure 11a. The peak current for the anodic oxidation of glucose is proportional to the square root of the scan rate, indicating a diffusion-controlled redox reaction.

Consistent with the principles governing many nanomaterials, small-scale structures possess a larger specific surface area, more active sites, and superior catalytic properties. The trend in sensitivity performance is closely tied to the morphological transformation of the plated layer. Increased current density leads to a general deterioration of the CTAB inhibition effect. Throughout this process, small structures on the electrode surface diminish, gradually evolving into large crystals and protuberances. At a current density of 0.75 ASD, the plating surface retains the beneficial small particle structures that enhance sensitivity. Simultaneously, the appearance of large and coarse crystals augments the electrode’s surface area, optimizing sensitivity through the combined effect of expanded surface area and small particles. As the current density continues to rise, the coating thickens and transforms into large crystals with poor catalytic properties, resulting in a decline in sensitivity. 

The performance details of the electrodes in this paper are summarized and presented in Table 2. Similar linear ranges are exhibited by all the electrodes; nonetheless, the plating layers, acquired through electroplating under various conditions, exerted a substantial and discernible influence on sensitivity. The observed enhancement in sensitivity, when compared to the bare electrode, underscores the pivotal role played by the plating process.

Moreover, this electrode’s performance is compared with that of other non-enzymatic sensors, and their respective electrode types and sensing materials are detailed in Table 3. It is evident that, in contrast to alternative sensors, the electrodes in this study exhibit an impressively sensitive performance. This superiority can be attributed to the expansive surface area of the micropillar array and the exceptional catalytic properties inherent in the plating structure.

### 3.5. Selectivity Evaluation of the Glucose Sensor

**Figure 12:**
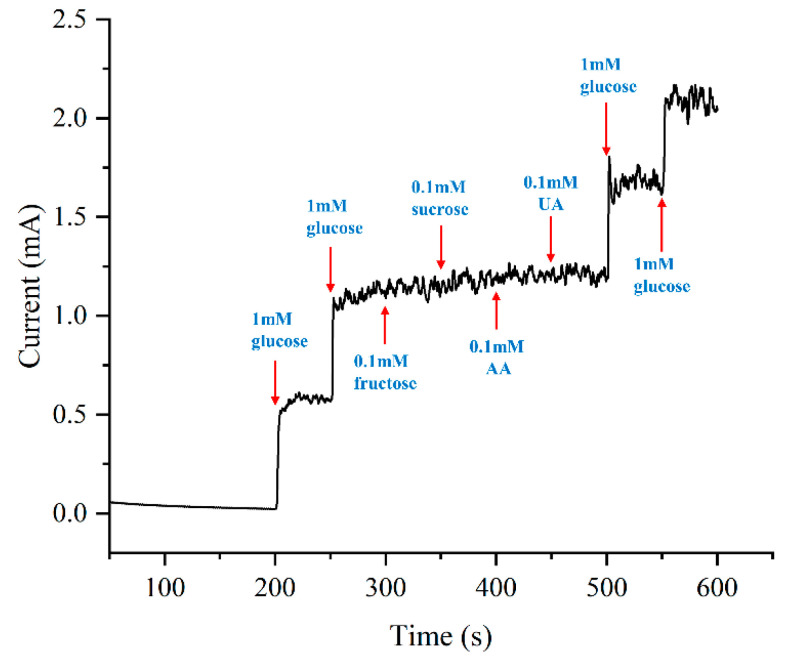
Selectivity test of the sample plated at 0.75 ASD.

Human serum contains a diverse array of organic compounds susceptible to oxidation at positive potentials, potentially introducing interference to the sensor. Therefore, several representative species such as fructose, sucrose, AA, and UA were deliberately chosen to assess the selectivity of the sensor, with concentrations referenced to physiological levels [38,39]. To establish a baseline, glucose was initially added to the solution twice, achieving a concentration of 2 mM. Subsequently, the test species were introduced sequentially at 50-s intervals, and Figure 12 presents the amperometric responses. 

Following the addition of glucose, the current of the electrode exhibits a rapid and substantial increase. In contrast, the electrode exhibits a negligible response to the four interfering species. To evaluate the extent of interference, the current data from the middle 30-s interval after species addition are averaged and compared with the data from the preceding period. The incremental changes in current are 3.67%, 3.51%, 2.54%, and 0.83%. All changes are less than 5%, underscoring the high selectivity of the electrode. The remarkable selectivity observed may be attributed to the structure of the micropillar arrays, characterized by a large surface area that leads to a more diffuse distribution of the electric field. The moderated electric field distribution, in turn, diminishes the likelihood of oxidation occurring for interfering species at the same potential.

## 4. Conclusions

In this study, micropillar array electrodes for glucose detection were meticulously crafted through standard microelectronics processes and electroplating. The investigation delved into the impacts of CTAB concentration and current density on plating morphology and detection performance. In the absence of CTAB during the electroplating process, the plating solution yielded a profusion of irregular crystals with challenging morphology distribution control. The introduction of CTAB as an inhibitor effectively mitigated protrusions, resulting in a uniform plating layer. Notably, an augmentation in CTAB concentration was observed to refine the grains, leading to an improvement in detection sensitivity. At a CTAB concentration of 1 g/L, a velvety plating layer was achieved, elevating the electrode sensitivity to 3409 μA mM^−1^ cm^−2^. The correlation between morphology and sensitivity is also explored.

Plating current density emerged as a pivotal factor influencing coating morphology and detection performance. As the current density increased, the plated surface transitioned from fine grains to large and coarse crystals, showcasing a sensitivity peak before diminishing. Optimal sensitivity and response current were attained at 3314 μA mM^−1^ cm^−2^ and 21.956 mA/cm^2^, respectively. This outcome is attributed to the synergistic effect of fine particles and enhanced surface area due to the rough surface.

Furthermore, the stability of the electrode has also been investigated. In a solution containing interfering species at a concentration of 0.1 mM, the electrode exhibited robust performance with negligible interference from the four measured species, demonstrating changes in current below 5%. This impressive selectivity underscores its potential for practical applications. 

In conclusion, the micropillar array electrodes, optimized through careful consideration of CTAB concentration and plating current density, presents a promising avenue for sensitive and stable glucose detection. The study provides valuable insights into the interplay between morphology and performance, paving the way for enhanced biomedical detection applications in the future. By employing standard microelectronic processes, the fabrication of the working electrode, counter electrode, and reference electrode on a single silicon chip is facilitated. This capability allows for the miniaturization of the sensor, thereby reducing the required amount of analyte. Furthermore, the integration of micropillar array electrodes into microflow chips holds the potential to augment their reaction area and overall performance.

## Figures and Tables

**Table 1:** Glucose addition details for CA tests.

Sequence	Concentration (μM)	Volume (μL)	Frequency
1	1	10	1
2	3	30	3
3	20	20	3
4	50	50	4
5	200	20	4
6	500	50	5
7	1000	10	4
8	2000	20	4

**Table 2:** Analytical performance of the electrodes plated under different conditions.

	Linear Range (mM)	R^2^	LOD (S/N = 3) (μM)	Sensitivity (μA mM^−1^ cm^−2^)
Bare	0.03–1.5	0.99717	10.1	2059
CTAB 0	0.03–1.5	0.99752	12.7	2101
CTAB 0.01	0.03–1.5	0.99662	6.6	2909
CTAB 0.1	0.03–1.5	0.9983	6.3	2985
CTAB 1	0.03–1.5	0.99744	7.4	3409
0.3 ASD	0.03–1.5	0.99559	9.3	2338
0.75 ASD	0.03–1.5	0.99864	15.9	3314
1.5 ASD	0.03–1.5	0.99689	13.7	3104
3 ASD	0.03–1.5	0.99648	15.0	2865

**Table 3:** Comparison of non-enzymatic glucose sensor in this work with similarly reported sensors.

Electrode	Sensing Material	Sensitivity (μA·mM^−1^·cm^−2^)	Linear Range (mM)	Detection Limit (μM)	Reference
GCE	Cu-Co sulfide microparticles	1475.97	0.001–3.66	0.1	[31]
copper foil	CuNPS-graphene	379.31	0.02–2.3	1.39	[32]
GCE	Cu-Pt NPs	2209	0.01–0.75	1.8	[33]
GCE	CuNi-MOFNs	702	0.01–4	3.33	[34]
graphite electrode	Cu@PCR	847	0.01–7.5	0.043	[35]
GCE	hollow spherical CuCo_2_O_4_	2929.4	2–1.8	0.27	[36]
LIG	Au/Ni layer	3500	0–30	1.5	[37]
Micropillar array	Cu	3409	0.03–1.5	7.4	This work

## Data Availability

Data are contained within the article.
